# SHP2 inhibitor protects AChRs from effects of myasthenia gravis MuSK antibody

**DOI:** 10.1212/NXI.0000000000000645

**Published:** 2019-12-12

**Authors:** Saif Huda, Michelangelo Cao, Anna De Rosa, Mark Woodhall, Pedro M. Rodriguez Cruz, Judith Cossins, Michelangelo Maestri, Roberta Ricciardi, Amelia Evoli, David Beeson, Angela Vincent

**Affiliations:** From the Department of Clinical Neurosciences (S.H., M.C., M.W., P.M.R.C., J.C., D.B., A.V.), Weatherall Institute of Molecular Medicine and Nuffield, University of Oxford, UK; Department of Clinical and Experimental Medicine (A.D.R., M.M., R.R.), Neurology Unit, Pisa; and Department of Neuroscience (A.E.), Catholic University, Rome, Italy.

## Abstract

**Objective:**

To determine whether an SRC homology 2 domain-containing phosphotyrosine phosphatase 2 (SHP2) inhibitor would increase muscle-specific kinase (MuSK) phosphorylation and override the inhibitory effect of MuSK-antibodies (Abs).

**Methods:**

The effect of the SHP2 inhibitor NSC-87877 on MuSK phosphorylation and AChR clustering was tested in C2C12 myotubes with 31 MuSK-myasthenia gravis (MG) sera and purified MuSK-MG IgG4 preparations.

**Results:**

In the absence of MuSK-MG Abs, NSC-87877 increased MuSK phosphorylation and the number of AChR clusters in C2C12 myotubes in vitro and in DOK7-overexpressing C2C12 myotubes that form spontaneous AChR clusters. In the presence of MuSK-MG sera, the AChR clusters were reduced, as expected, but NSC-87877 was able to protect or restore the clusters. Two purified MuSK-MG IgG4 preparations inhibited both MuSK phosphorylation and AChR cluster formation, and in both, clusters were restored with NSC-87877.

**Conclusions:**

Stimulating the agrin-LRP4-MuSK-DOK7 AChR clustering pathway with NSC-87877, or other drugs, could represent a novel therapeutic approach for MuSK-MG and could potentially improve other NMJ disorders with reduced AChR numbers or disrupted NMJs.


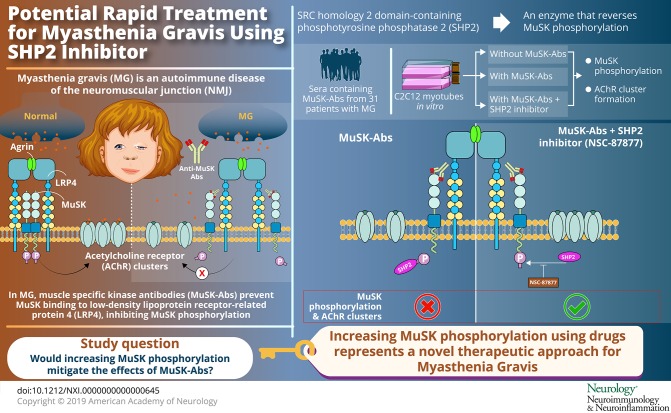


Myasthenia gravis (MG) is an autoimmune disease of the neuromuscular junction (NMJ). In a variable percentage of patients, muscle-specific kinase antibodies (MuSK-Abs) are present.^1^ In humans, symptoms occur particularly in cranial, bulbar, and respiratory muscles with frequent respiratory crises.^2^ Although immunomodulatory treatments can be beneficial in the longer term, there is an unmet need for cheap, fast, and effective treatments.

MuSK is normally activated following binding of agrin, secreted from the motor nerve terminal, to low-density lipoprotein receptor-related protein 4 (LRP4). LRP4 then binds to MuSK leading to its dimerization and to auto- and trans-phosphorylation in the MuSK juxtamembrane region.^3^ The subsequent recruitment of intracellular downstream of kinase 7 (DOK7) further stimulates MuSK phosphorylation, causing activation of a phosphorylation cascade that ultimately leads to clusters of acetylcholine receptor (AChR) anchored by intracellular rapsyn on the postsynaptic membrane of the NMJ.^4^

MuSK-Abs are predominantly of the IgG4 subclass and inhibit the interaction between LRP4 and MuSK, preventing MuSK phosphorylation and AChR clustering.^5,6^ Considering this mechanism, increasing MuSK phosphorylation could represent a potential therapeutic strategy for the development of specific treatments.

Among several regulators of the AChR clustering pathway, the SRC (Rous sarcoma gene) homology 2 domain-containing phosphotyrosine phosphatase 2 (SHP2) is a phosphatase that reduces MuSK phosphorylation. Importantly, for the purposes of this study, NSC-87877, an SHP2 inhibitor, has been shown to enhance agrin-induced and agrin-independent AChR clustering in vitro.^7^ We tested NSC-87877 for its ability to increase MuSK phosphorylation and to reverse or prevent the effects of MuSK-Abs in 2 in vitro models.

## Methods

### Materials

Serum samples were collected, with informed consent, from 31 patients with typical MuSK-MG symptoms and immunotherapy responses. MuSK-Ab titers of patients' sera and purified immunoglobulin G (IgG) fractions were determined by radioimmunoassay and cell-based assay (CBA).^8,9^ Sera were heated, dialyzed, and sterile filtered before use. IgG fractions were purified from plasmapheresis of 2 additional patients with MuSK-MG using protein G sepharose and an IgG4 affinity matrix.^5,8^ Effectiveness of IgG subclass purification was tested with CBA. Briefly, MuSK-transfected human embryonic kidney cells were incubated with the different IgG subclasses (1:20) and probed with anti-human-IgG1, -IgG2, -IgG3, and -IgG4 monoclonal mouse antibodies (1:50) (I2513, I5635, I7260, and I7385, Sigma). After fixing with 3% formaldehyde, cells were stained with Alexa Fluor 488 goat anti-mouse IgG (1:200) (A32723, Invitrogen) and images captured using the Olympus IX71 fluorescence microscope with Simple PCI (Digital Pixel). The SHP2 inhibitor NSC-87877 (#2613) was obtained from Tocris Bioscience, Bristol, United Kingdom.

### C2C12 myotube cultures and AChR cluster analysis

C2C12 mouse myoblasts (91031101, ATCC) were maintained and differentiated into myotubes after 5–6 days in differentiation medium as previously reported (Dulbecco’s modified Eagle medium [DMEM] with 2% fetal calf serum/horse serum).^9^ MuSK-MG sera with a broad range of MuSK-Abs (nM) were chosen according to availability. MuSK-MG sera (1:10, 1:30, and 1:90) or purified MuSK-Ab subclasses (0.5 nM) were applied to myotubes for 30 minutes and then incubated overnight with agrin 1:800 (short rat form, producing approximately 50% of maximum AChR clusters) in the presence and absence of NSC-87877 100 μM. AChR clusters were then labeled with Alexa Fluor 594 α-bungarotoxin (1:1,000) (B13422, Invitrogen) and fixed in 3% formaldehyde. Twenty fields selected with bright field were analyzed for number and cluster length (>3 μm) using ImageJ software.^8–11^

The *Musk* “knockout” (KO) C2C12s were generated with the clustered regularly interspaced short palindromic repeats (CRISPR)-Cas9 system using standard methods.^12^ Briefly, *Musk* guide oligonucleotides (Integrated DNA Technologies) were designed and cloned into a modified plasmid pX335-U6-Chimeric_BB-CBh-hSpCas9n-D10A (42335, Addgene). Guide A oligonucleotide sequences were A-forward: 5′-CACCGCATTCTCCCGGATGCTGTAG-3′ and A-reverse: 5′-AAACCTACAGCATCCGGGAGAATGC-3′. Guide B oligonucleotide sequences were B-forward: 5′-CACCGCTCCTCACCATTCTGAGCG-3′ and B-reverse: 5′-AAACCGCTCAGAATGGTGAGGAGC-3′. Myoblasts were electroporated with 10 μg of each plasmid using the Neon Transfection System (Life Technologies), selected with Geneticin Antibiotic (10131035, Life Technologies), and cloned in 96-well plates using fluorescence-activated cell sorting. Clones were screened by Sanger sequencing after PCR of genomic DNA using the primers 5′-TGGTGCTTTGGTTATGGAGCC-3′ and 5′-GAGGAGGGGTCTAAGGCTTG-3′. *Musk* KO generation was confirmed by Western blot.

DOK7-overexpressing myoblasts were prepared as previously described.^10,11^ The myotubes were exposed to MuSK-Ab or control sera or 0.5 nM MuSK-Ab of each purified preparation (IgG or IgG4) as illustrated in the figures.

### MuSK phosphorylation

MuSK phosphorylation was assessed by Western blotting on immunoprecipitates from the C2C12 myotubes. They were starved of fetal calf or horse serum for 3 hours, incubated in medium alone or medium plus NSC-87877 (1–1,000 µM; usually 100 µM) for 40 minutes at 37°C, lysed in cold lysis buffer (10 mM Tris-HCl, 1 mM EDTA, 100 mM, NaCl, 1% Triton X-100, 1× protease [P8340, Sigma] and phosphatase [78420, Thermo Fisher] inhibitor cocktails), and centrifuged. The supernatant was immunoprecipitated with anti-MuSK-Ab (AF562, Bio-Techne, Minneapolis, MN) using Protein G Dynabeads (10004D, Thermo Fisher) or directly with patients' MuSK-Abs for experiments with purified IgG and IgG4. Immunoprecipitated proteins were eluted into sodium dodecyl sulphate sample buffer, and the Western blots probed with anti-phosphotyrosine antibody 1:1,000 (4G10, Upstate Biotechnology, MA). The nitrocellulose was then stripped and reprobed with AF562. Band densitometry was performed using ImageJ software. MuSK phosphorylation levels were normalized to levels of immunoprecipitated MuSK.

### Statistical analysis

Statistical tests were performed with GraphPad Prism 6 as indicated in the figure legends. Error bars represent the standard error of the mean unless otherwise stated. Differences with *p* values <0.05 were considered statistically significant.

### Standard protocol approvals, registrations, and patient consents

The study of archived patient samples was approved by the Oxfordshire Research Ethics Committee A (07 Q160X/28).

### Data availability

The authors confirm that all the data supporting the findings of this study will be made available on reasonable request to the corresponding author.

## Results

### SHP2 inhibition increased MuSK phosphorylation and induced agrin-independent AChR clusters

**Figure 1:**
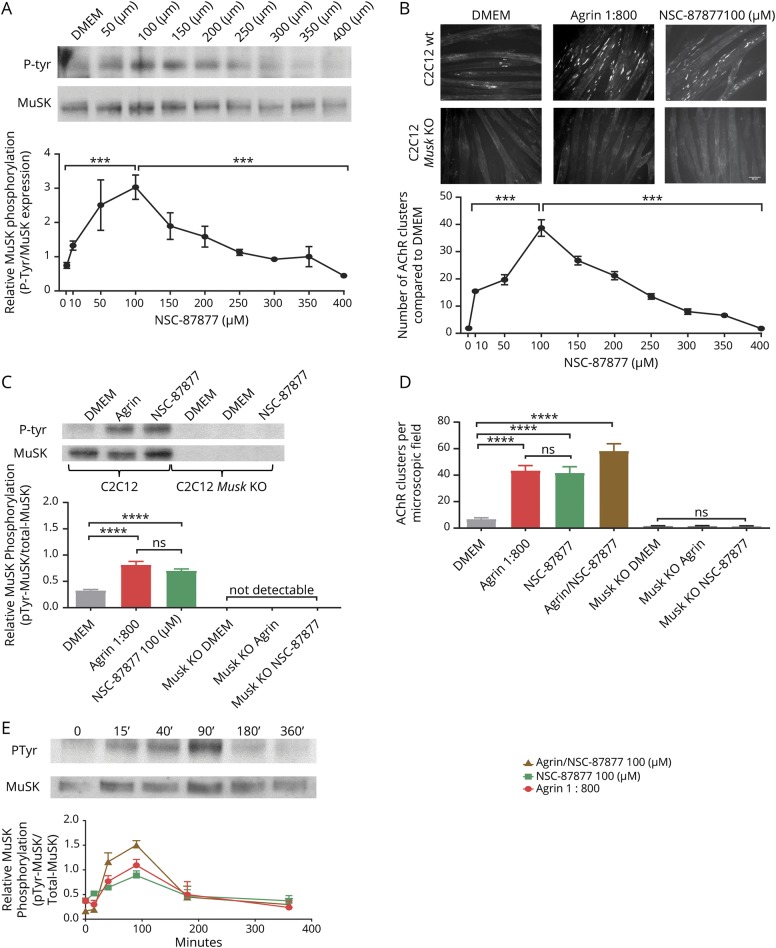
SHP2 inhibition by NSC-87877 induces MuSK phosphorylation and AChR clustering (A) Dose-response curve of the effects of SHP2 inhibition on AChR phosphorylation. Phosphorylation increased progressively reaching a maximal effect at 100 µM and decreasing at higher concentrations. (B) Clustering of AChRs (labeled with Alexa Fluor 594 α-bungarotoxin) in C2C12 myotubes after 12 hours incubation with DMEM, agrin, or 100 μM NSC-87877. Both agrin and NSC-87877 increased AChR clusters in native C2C12 myotubes but not in *MuSK* knockout cells. The optimal NSC-87877 concentration was 100 µM, and cluster numbers decreased at higher concentrations. (C and D) The pooled results of 3–6 independent experiments measuring MuSK phosphorylation (C) and AChR clusters (D) are shown. (E) Time course of MuSK phosphorylation in C2C12 myotubes after exposure to agrin (1:800), NSC-87877 (100 μM), and agrin/NSC-87877. In each case, a similar time course was observed with maximum effect between 40 and 90 minutes. Phosphorylation and MuSK blots shown for NSC-87877 alone. All images are 20× magnification. Scale bar represents 50 µm. Mean + standard error of the mean are shown. Results were analyzed by 2-sided *t* tests. AChR = acetylcholine receptor; MuSK = muscle-specific kinase; SHP2 = SRC homology 2 domain-containing phosphotyrosine phosphatase 2.

First, we studied the effect of SHP2 inhibition on MuSK phosphorylation and on AChR clustering in the absence of agrin. We exposed C2C12 myotubes to increasing concentrations of NSC-87877 (1–400 µM) for 40 minutes. MuSK was immunoprecipitated by commercial anti-MuSK AF562. Phosphorylation was identified in the immunoprecipitates by Western blotting with an anti-tyrosine phosphorylation antibody and MuSK expression by incubating the stripped membranes with anti-MuSK AF562. AChR clusters were measured on parallel myotube cultures by labeling with fluorescent alpha-bungarotoxin after 12 hours. The ratio of phosphorylated MuSK to total MuSK increased substantially (each normalized to that in DMEM alone) with maximal effect at 100 µM; at higher concentrations, phosphorylation decreased (figure 1A). C2C12 myotubes, in the absence of agrin, express few spontaneous clusters (<10 per field; figure 1B DMEM), but after incubation with agrin, the numbers increased up to 40/field. As previously shown,^7^ NSC-87877 at 100 µM, without agrin, increased the numbers of AChR clusters similar to those achieved with agrin alone (figure 1B upper panel). Again, the optimal concentration was 100 µM, which was used for all experiments thereafter. As expected, neither MuSK nor phosphorylated MuSK was detectable in the *Musk* KO myotubes (figure 1B, lower panel). Mean results of different experiments are shown in figures 1, C and D.

To ensure that 40 minutes was appropriate for the experiments, a time course was performed. This showed that MuSK phosphorylation increased over time and was clearly detectable at 40 and 90 minutes for agrin, NSC-87877, or both NSC-87877 and agrin. MuSK phosphorylation was modestly increased when both were given together compared with NSC-87877 alone (*p* < 0.05 at 40′, *p* < 0.01 at 90′) (figure 1E), and the submaximal 40 minutes was chosen for future experiments looking at changes in MuSK phosphorylation.

**Figure 2:**
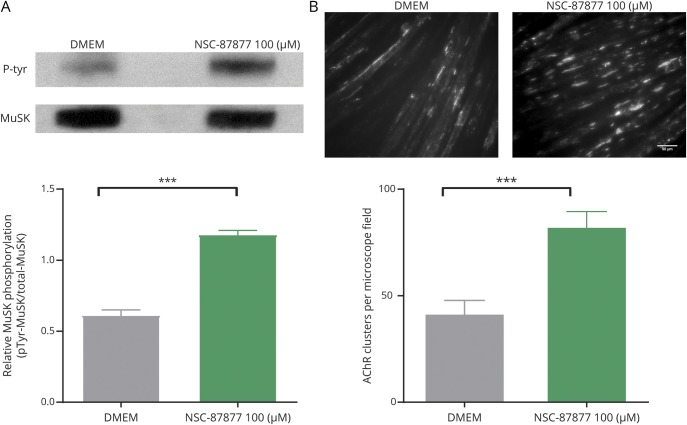
Effect of SHP2 inhibition in DOK7-overexpressing C2C12s (A) MuSK phosphorylation was already high in DOK7-overexpressing C2C12s (data not shown) but was increased further by NSC-87877. (B) Equally DOK7-overexpressing myotubes showed AChR clusters that were further increased by NSC-87877. All images are 20× magnification. Scale bar represents 50 µm. Results were analyzed by 2-sided t tests. DOK7 = downstream of kinase 7; MuSK = muscle-specific kinase; SHP = SRC homology 2 domain-containing phosphotyrosine phosphatase 2.

C2C12 myotubes overexpressing DOK7 develop stable AChR clusters in the absence of agrin.^11^ MuSK phosphorylation was clearly present in the DOK7-overexpressing cells without added agrin and was further increased by NSC-87877 (figure 2A). Moreover, the addition of NSC-87877 to the DOK7-overexpressing myotubes increased the number of AChR clusters that mirrored the increased phosphorylation (figure 2B).

### Patients with MuSK-MG

To investigate whether SHP2 inhibition was able to protect against the effects of MuSK-Abs, 31 MuSK-MG sera (female:male, 9:1; median age at onset 42 years [range 18–70 years]) were studied. All patients except 3 were on immunotherapies, mainly prednisolone and/or azathioprine, but all had predominantly bulbar disease (as indicated by b grades), and 8 still had severe involvement (Myasthenia Gravis Foundation of America 3b or 4b, see table). MuSK-Ab levels measured by radioimmunoprecipitation were typically variable (0.25–24.26 nM). CBAs showed that, as expected, IgG4 MuSK-Abs were present and predominated over IgG1, 2, or 3 (data not shown).

**Table:**
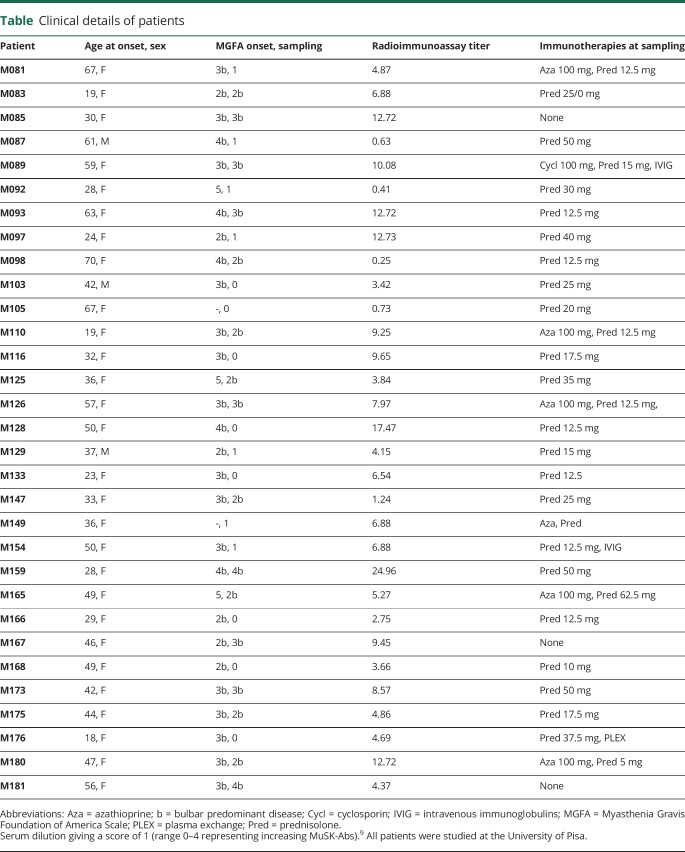
Clinical details of patients

### SHP2 inhibition alleviates the effect of MuSK-Abs on agrin-induced and agrin-independent AChR clusters

**Figure 3:**
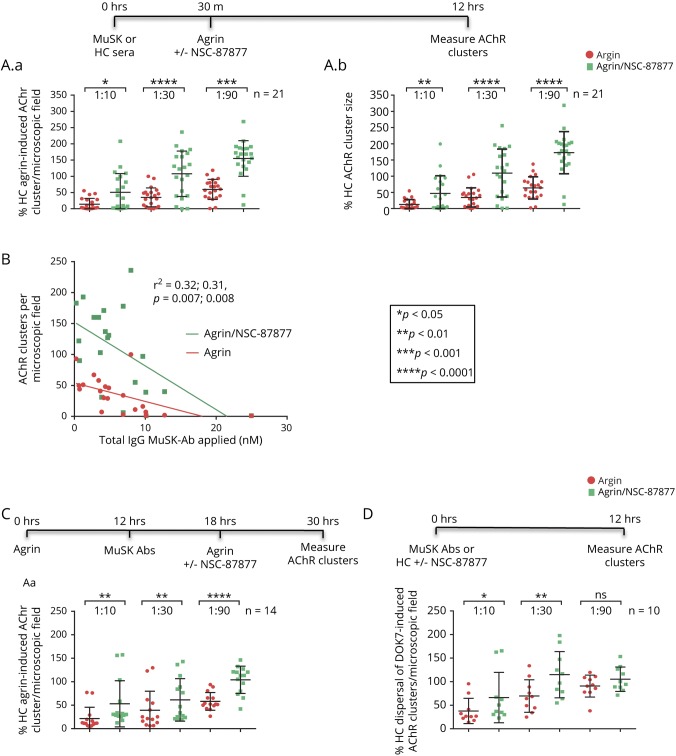
SHP2 inhibition reverses effects of MuSK-Abs on AChR clusters in C2C12s and DOK7-overexpressing C2C12s (A) The treatment protocols are shown above the results. MuSK-Ab positive (n = 21) or healthy control sera (n = 2) were incubated with myotubes for 30 minutes, followed by agrin (1:800) with or without NSC-87877 (100 μM). After 12 hours, the AChR clusters were analyzed and expressed as a percentage of control serum results. The number (A.a) of the AChR clusters without NSC-87877 was clearly reduced by the MuSK-Abs (to 14%, 35%, and 60% at dilutions 1:10, 1:30, and 1:90, red columns); the size of the clusters was similarly reduced (A.b). In each case, the effects were partially or completely reversed by NSC-87877 (green columns). (B) Inverse correlation between the number of AChR clusters at 1:30 serum dilution and MuSK-Ab titers (nM) with or without NSC-87877. (C) Using a different protocol, AChR clusters were similarly reduced, and there was partial or complete protection by NSC-87877. (D) The effects of the different serum dilutions on AChR clusters and the protection by NSC-87877 were also seen in the DOK7-overexpressing C2C12s, although to a lesser extent. In A, C, and D, comparisons at each different serum concentration were analyzed by 2-sided *t* tests. For B, linear regression was computed by GraphPad Prism. The scatter plots show results of individual sera, with mean ± SDs. AChR = acetylcholine receptor; MuSK = muscle-specific kinase; SHP2 = SRC homology 2 domain-containing phosphotyrosine phosphatase 2.

Three dilutions (1:10, 1:30, and 1:90) of 21 MuSK-Abs and 2 control sera were incubated with the myotubes for 30 minutes before adding agrin with or without NSC-87877; 12 hours later, the numbers of AChR clusters were measured (see time line in figure 3A). In each case, the results were expressed as % of those in the presence of healthy control serum dilutions with agrin but without the SHP2 inhibitor. MuSK-MG sera reduced the number and size of AChR clusters with most effect at 1:10 dilution as expected and, at each serum concentration, NSC-87877 (100 µM) increased the number and size of AChR clusters (figure 3, A.a and A.b). There was a modest correlation between the number of clusters at 1:30 serum dilution and MuSK-Ab radioimmunoprecipitation titers, with and without NSC-87877 (figure 3B), with similar results or trends at 1:10 and 1:90 (data not shown).

When SHP2 inhibitor and agrin were added 6 hours after the MuSK-Abs to see whether the inhibitor could recluster the AChRs following dispersal, the numbers of clusters were again increased at each serum dilution (figure 3C).

On the DOK7-overexpressing myotubes, where the clusters are well established, MuSK-MG sera reduced the number of AChR clusters but to a lesser extent than the same sera on agrin-induced AChR clusters. At 1:30 serum dilutions, for instance, the number of agrin-induced clusters was 27.9% ± 9.3% (n = 10) of control, whereas on DOK7-overexpressing myotubes, the number of clusters was 69.6 ± 10.9%, n = 10; *p* = 0.01) of control-treated myotubes. Nevertheless, NSC-87877 increased the number of clusters (figure 3D) at 1:10 and 1:30 dilutions. At 1:90 dilution, the sera did not significantly reduce the number of clusters, and NSC-87877 had no effect.

### SHP2 inhibition increases AChR clusters in IgG4-treated C2C12 myotubes

**Figure 4:**
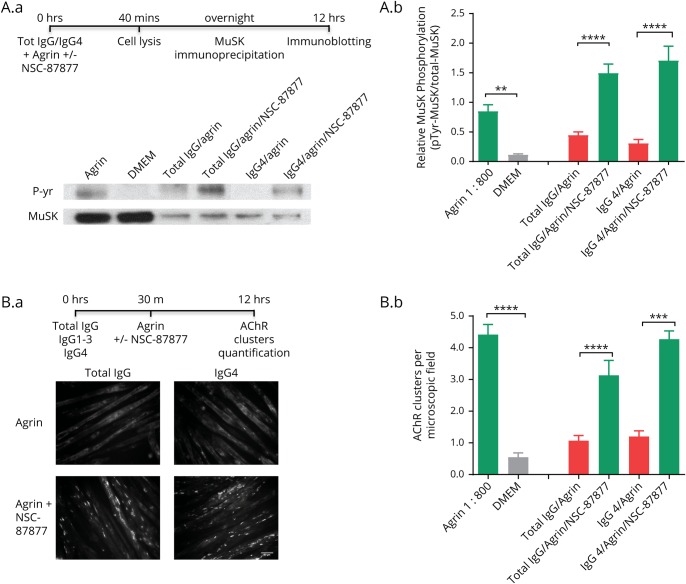
IgG and IgG4 subclass MuSK-Abs reduce AChR clusters, which are restored by NSC-87877 Agrin-stimulated myotubes were exposed for 40 minutes to MuSK IgG or IgG4 subfraction (adjusted to 0.5 nM MuSK-Ab) purified from 2 patients with MuSK-MG (the total number of experiments was IgG n = 5; IgG4 n = 6). (A.a) Example of phosphorylation blots. MuSK was immunoprecipitated by anti-MuSK AF562 or MuSK-MG IgG fractions. Pooled results (A.b) show that total IgG and IgG4 inhibited MuSK phosphorylation, and this was increased substantially by NSC-87877 (100 μM). (B.a) Examples of AChR clusters. Pooled results (B.b) show that total IgG and Ig4 MuSK-Ab fractions inhibited the formation of AChR clusters, which in each case were increased by NSC-87877. Two-sided *t* tests were used for comparisons at each IgG concentration. AChR = acetylcholine receptor; MG = Myasthenia gravis; MuSK = muscle-specific kinase.

Finally, to demonstrate that the effects were due (at least mainly) to IgG4 MuSK-Abs, purified MuSK-Ab IgGs and IgG4 subfractions were prepared from plasmapheresis material of 2 additional patients with typical MuSK-MG. For myotubes not exposed to patient antibodies, MuSK was immunoprecipitated by the anti-MuSK AF562 antibody from the cell lysates (to avoid MuSK phosphorylation with concomitant use of the commercial antibody). For myotubes exposed to patient antibodies, to ensure that only phosphorylation of the antibody-bound MuSK was assessed, MuSK was immunoprecipitated by the patient IgG. To adjust for this difference in the precipitation technique, the phosphorylation signals were always normalized to the MuSK protein signal. An example immunoblot is shown in figure 4, A.a and results of 3–6 experiments in figure 4, A.b. Both total MuSK-MG IgG and IgG4 fractions reduced MuSK phosphorylation, and phosphorylation was enhanced by NSC-87877 (figure 4, A.a and A.b).

Figure 4, B.a and B.b show the effects on AChR clusters. Again, both total IgG and IgG4 preparations substantially reduced agrin-induced AChR clustering, as previously reported.^8^ Moreover, in each case, NSC-87877 protected the myotube clusters from the inhibitory effects.

## Discussion

MuSK-MG can be a life-threatening disease that responds poorly to symptomatic drugs such as cholinesterase inhibitors and often requires high doses of steroids and other immunosuppressive drugs for long-term treatment. Patients may have myasthenic crises with respiratory and bulbar involvement that often require urgent treatment in intensive care. The development of a therapy that acts specifically on the mechanisms of the disease could achieve quick control of symptoms and in the longer term might help reduce the potential side effects from immunosuppressive therapies. Thus, targeting the intracellular regulation of the AChR clustering pathway by inhibition of the specific phosphatase, SHP2, as we show here, could represent a novel and specific therapeutic strategy for MuSK-MG. The results also draw attention to the potential relevance of targeting intracellular pathways in diseases caused by extracellular antibodies or other mechanisms.

There are several proteins downstream of MuSK that could be potential pharmacologic targets, including Abl tyrosine kinases and guanosine triphosphatases of the Rho family. However, increasing the activity of these regulators to enhance AChR clustering could lead to tumor development as many of them, such as ErbB2 and mitogen-activated protein kinase/extracellular-signal-regulated-kinase,^13,14^ play an important role in cell growth. By contrast, Src homology 2 domain-containing phosphatase 2 (SHP2), encoded by the *PTPN11* gene, is a potentially safer target. Under physiologic conditions, through a negative-feedback loop, SHP2 reduces MuSK phosphorylation, providing homeostatic control of AChR clusters at the NMJ. SHP2 also enhances cell replication inducing different pathways such as Ras/Erk, PI3K, serine/threonine kinase 1, and janus kinase 2-signal transducer and activator of transcription^15–17^; thus, SHP2 inhibition should not increase the risk of tumorigenesis. Indeed, pharmacologic inhibition of SHP2 has already demonstrated efficacy in the treatment of a mouse glioma xenograft model.^18^

The selective inhibition of the SHP2 phosphatase that controls MuSK phosphorylation by NSC-87877 was shown previously to increase AChR clustering in C2C12 myotubes^7^; here, we confirm that this works through increasing phosphorylation, which was clearly seen irrespective of the presence of agrin or MuSK-Abs. Although NSC-87877 is known to act on both SHP1 and SHP2, only SHP2 has been detected in C2C12 myotubes,^7,19^ and, reassuringly, conditional knockout of SHP2 in skeletal muscle did not compromise the formation and maintenance of the NMJ, suggesting that pharmacologic inhibition of SHP2 should be well tolerated in skeletal muscle,^20^ although other SHP2-specific inhibitors should also be studied.

MuSK-Abs are mainly IgG4 subclass and are known to inhibit MuSK activation and phosphorylation by blocking the interaction between LRP4 and MuSK.^5,8^ The inhibitory effects of MuSK-Abs on AChR clustering were present to varying degrees in all sera studied, correlating broadly with MuSK-Ab titers. NSC-87877 improved phosphorylation and AChR clustering at each concentration of the sera tested. Moreover, when the phosphorylation and AChR clustering experiments were conducted comparing total IgG and the IgG4 subfraction from 2 patients, both reduced MuSK phosphorylation and AChR clustering, and SHP2 inhibition by NSC-87877 was able to reverse their pathologic effects. Of interest, the inhibitory effect of IgG4 on AChR clustering does not depend on its monovalency and divalent monoclonal antibodies to MuSK also inhibit clustering.^21,22^ These recent findings suggest that MuSK antibodies could have additional pathogenic mechanisms, other than the direct inhibition of agrin/MuSK interaction, which contribute to the disruption of the AChR clustering pathway. Nevertheless, the inhibition of MuSK phosphorylation still remains the main effect of MuSK antibodies, as confirmed by our results with purified total IgG and by the effectiveness of the inhibition of SHP2.

There were some limitations to the experimental procedure. For the phosphorylation experiments, the patient antibody-precipitated MuSK was less than the MuSK precipitated by commercial antibody from the control cultures. There were 2 reasons for this. First, we chose a concentration of patient MuSK antibody (0.5 nM) that was submaximal for inhibition of clustering, making sure that any effects on clustering were clearly evident, but we needed to ensure that we only measured the phosphorylation in those MuSK molecules that were bound by patient IgG. Second, the commercial antibody was applied to the cell lysate from the myotubes rather than to the live cells and thus could also precipitate MuSK that was not on the surface. To adjust for these differences in MuSK immunoprecipitation, the phosphorylation was normalized to the MuSK protein signal for each lane. Further in vitro studies examining the intracellular molecular mechanisms need to be performed, and the protective effects of SHP2 inhibition against MuSK antibodies demonstrated in vivo.

Most novel therapies in MG depend on monoclonal antibodies targeting the immune system, but given the understanding of the mechanisms involved in the MuSK pathway, faster pharmacologic treatments should be explored. Recently, a monoclonal MuSK antibody was shown to stimulate MuSK phosphorylation and to preserve NMJs in a motor neuron disease model.^23^ Using new or repurposed drugs to enhance MuSK phosphorylation, or other downstream components of the signaling pathway, could be beneficial in MuSK-Ab myasthenia and perhaps in other disorders of neuromuscular transmission where loss of AChRs or disruption of the NMJ structure underlies the muscle weakness.

## Glossary

AChR: acetylcholine receptor
CBA: cell-based assay
DOK7: downstream of kinase 7
FACS: fluorescence-activated cell sorting
KO: knockout
LRP4: low-density lipoprotein receptor-related protein 4
MG: myasthenia gravis
MuSK: muscle-specific kinase
NMJ: neuromuscular junction
SHP2: Src homology 2 domain-containing phosphotyrosine phosphatase 2
SK: specific kinase
